# Whole-Genome Analysis of *Mycobacterium tuberculosis* from Patients with Tuberculous Spondylitis, Russia

**DOI:** 10.3201/eid2403.170151

**Published:** 2018-03

**Authors:** Ekaterina Chernyaeva, Mikhail Rotkevich, Ksenia Krasheninnikova, Andrey Yurchenko, Anna Vyazovaya, Igor Mokrousov, Natalia Solovieva, Viacheslav Zhuravlev, Piotr Yablonsky, Stephen J. O’Brien

**Affiliations:** St. Petersburg State University, St. Petersburg, Russia (E. Chernyaeva, M. Rotkevich, K. Krasheninnikova, A. Yurchenko, P. Yablonsky, S.J. O’Brien);; St. Petersburg Research Institute of Phthisiopulmonology, St. Petersburg (E. Chernyaeva, N. Solovieva, V. Zhuravlev, P. Yablonsky);; University of Glasgow, Glasgow, Scotland, UK (A. Yurchenko);; St. Petersburg Pasteur Institute, St. Petersburg (A. Vyazovaya, I. Mokrousov);; Nova Southeastern University, Ft. Lauderdale, Florida, USA (S.J. O’Brien)

**Keywords:** *Mycobacterium* *tuberculosis*, tuberculous spondylitis, whole-genome sequencing, WGS, phylogeny, genetic markers, genome determinants, bacterial virulence, pathogenicity, drug resistance, Russia, bacteria, antimicrobial resistance, tuberculosis and other mycobacteria

## Abstract

Whole-genome analysis of *Mycobacterium tuberculosis* isolates collected in Russia (N = 71) from patients with tuberculous spondylitis supports a detailed characterization of pathogen strain distributions and drug resistance phenotype, plus distinguished occurrence and association of known resistance mutations. We identify known and novel genome determinants related to bacterial virulence, pathogenicity, and drug resistance.

Tuberculosis (TB) is an infectious disease caused by *Mycobacterium tuberculosis*, which typically affects the lungs but can affect other sites. In 2016, an estimated 10.4 million new TB cases and 1.6 million TB-related deaths were documented worldwide (*1*). The Russian Federation reported >120,000 TB cases and ≈13,700 TB deaths in 2016 (*1*). TB strains with multidrug resistance (MDR TB), characterized by resistance to isoniazid or rifampin, are common in the Russian Federation. The estimated rate of MDR TB was 27% among TB case-patients newly diagnosed in 2016 and 65% among previously treated case-patients in 2016 (*1*). Most TB cases are associated with pulmonary localization of the disease; however, in some cases, extrapulmonary TB develops. In Russia, the rate of extrapulmonary TB cases among new TB cases was 3.3% in 2014; most extrapulmonary TB cases are osteoarticular and genitourinary (*2*). Approximately 70% of osteoarticular TB cases are tuberculous spondylitis (TBS), which cause severe specific lesions of >1 components of the spine (*2*). We report whole-genome sequencing (WGS) and variant analyses of *M. tuberculosis* isolates from patients treated in Russia for TBS during 2007–2014. 

## The Study

**Figure 1:**
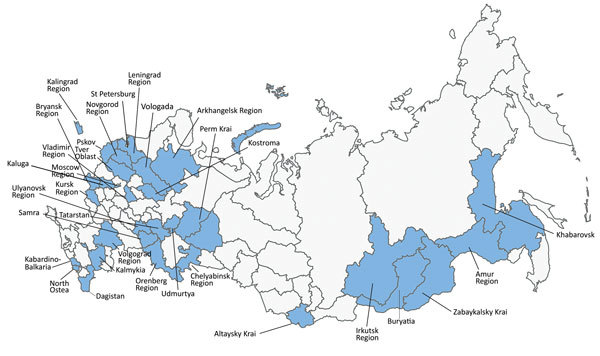
Distribution of *Mycobacterium* *tuberculosis* isolates randomly collected from 71 patients with tuberculous spondylitis who received treatment at clinics of Research Institute of Phthisiopulmonology in 32 regions of the the Russian Federation, 2007–2014

The isolates were randomly collected from 71 TBS patients who received treatment at clinics of the Research Institute of Phthisiopulmonology in 32 regions of the Russian Federation (Figure 1). In these cases, *M. tuberculosis* isolates were cultured from extrapulmonary clinical material and stored. We assessed the susceptibility of these stored isolates to streptomycin, isoniazid, rifampin, ethambutol, pyrazinamide, ethionamide, ofloxacin, kanamycin, amikacin, cycloserine, capreomycin, and paraaminosalicylic acid according to World Health Organization recommendations (*3*). We isolated genomic DNA from cultured bacteria by using phenol/chloroform extraction and subjected bacterial DNA to WGS by using the MiSeq platform (Illumina, San Diego, CA, USA) to a mean coverage of 47× (range 18×–170×), covering ≥99% of the *M. tuberculosis* H37Rv reference genome (GenBank accession no. NC_000962.3). We deposited WGS reads in the NCBI Sequence Read Archive (accession no. PRJNA352769). 

We aligned sequenced reads to the reference genome and called variants (single-nucleotide polymorphisms [SNPs] and short insertions/deletions) by using bioinformatics software: bowtie2 (http://bowtie-bio.sourceforge.net/bowtie2/index.shtml); SAMtools (http://samtools.sourceforge.net); VCFtools (http://vcftools.sourceforge.net); and FreeBayes (https://github.com/ekg/freebayes). We used mutations that had q-scores ≥20 for comprehensive analysis. We used concatenated SNPs for phylogenetic analysis by using the GTRCAT (general time-reversible model with rate heterogeneity accommodated by using discrete rate categories) maximum-likelihood algorithm from the RAxML software package (*4*) to calculate an approximation model and 100 bootstrap replications. To avoid misalignments, we annotated SNPs in repetitive genome regions and in genes encoding proteins that contain proline-glutamate or proline-proline-glutamate motifs and filtered them from analysis. We used PhyTB (*5*) and SpoTyping tools (*6*) for phylogenetic classification of *M. tuberculosis* genomes and verified SpoTyping output by using previously conducted conventional spoligotyping analysis for 20 isolates that were previously described (*7*).

**Figure 2:**
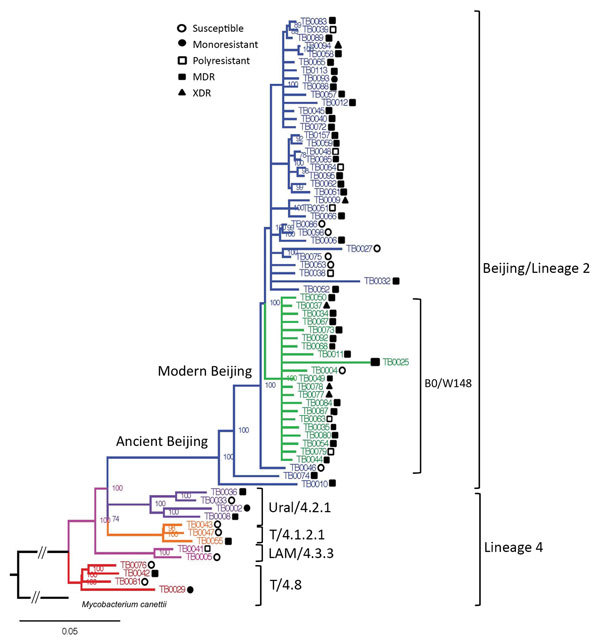
Phylogenetic analysis of *Mycobacterium* *tuberculosis* isolates obtained from patients with tuberculous spondylitis, Russian Federation, 2007–2014. Symbols indicate drug resistance: susceptible to all tested drugs; monoresistant to 1 drug; polyresistant, resistant to multiple drugs other than isoniazid or rifampin; MDR, resistant to isoniazid or rifampin; XDR, resistant to isoniazid or rifampin plus any fluoroquinolone and >1 of 3 injectable second-line drugs. Number of isolates and parsimony informative sites by genetic group: All groups, 71/2,656; Beijing, 58/919; Beijing B0/W148, 21/213; Beijing non-B0/W148, 37/722; T (4.8), 4/38; Ural, 4/204. The truncated root branch connects the studied *M. tuberculosis* lineages with an outgroup represented by *M. cannetii*. Scale bar indicates nucleotide substitutions per site.

We identified 2 principal phylogenetic lineages among *M. tuberculosis* isolates, lineage 2 and lineage 4; further, we detected evolutionary ancient and modern sublineages within major lineage 2 (Beijing; Figure 2) according to previously described classifications (*8*). The largest subgroup within the Beijing clade belonged to the B0/W148 clonal cluster (Figure 2) (*8*). Lineage 4 was represented by 4 genetic families: Ural, 4.2; Latin-American/Mediterranean (LAM),(4.3); and T, 4.1 and 4.8. The 58 Beijing genotype isolates contained 38 MDR (65.5%) 5 extensively drug-resistant (XDR; resistant to isoniazid and rifampin plus any fluoroquinolone and >1 of 3 injectable second-line drugs) (8.6%), 7 polyresistant but not MDR (12%), 1 monoresistant (1.9%), and 7 susceptible (12%) TB isolates. The MDR TB frequency in the Beijing group (65.5%), was higher than that for other genetic groups pooled (p<0.0096 by Fisher exact test). The *M. tuberculosis* Beijing B0/W148 cluster was represented by 1 susceptible (4.8%), 2 polyresistant (9.5%), 3 XDR (14.3%), and 15 MDR (71.4%) TB isolates. The B0/W148 genetic group demonstrates an association with MDR TB (p = 0.03), shown previously (*9*,*10*). The other genetic groups (T, LAM, and Ural) included too few isolates to test for association with MDR TB.

Specimens of 50 TBS patients were HIV negative; 21 were HIV positive. Although we found no significant association of *M. tuberculosis* genetic groups to HIV infection, 42% of patients infected by B0/W148 strains were HIV positive whereas among patients infected by non-B0/W148 Beijing strains, only 22% were HIV positive. Further, only 14% patients infected with non-Beijing *M. tuberculosis* strains were HIV positive (Technical Appendix Table 1).

We examined *M. tuberculosis* isolates for the presence of published variants associated with resistance to TB drugs (Table). We found a high level of concordance of phenotypic and genetic data for reported isoniazid- and rifampin-resistant isolates. We detected mutations in *rpsL*, *gid*, and *rrs* genes in 96.4% of streptomycin-resistant isolates; 81.8% of ofloxacin-resistant isolates had mutations in *gyrA* gene (there were no mutations in *gyrB* gene). Most ethambutol-resistant isolates (72.7%) showed mutations in the *embA* promoter region or *embB* region between codons 296 and 497. However, 3 ethambutol-susceptible isolates had mutations M306I (n = 1) and G406A (n = 2) in the *embB* gene. We detected mutations in genes *pncA* (*11*) and *rpsA*, associated with pyrazinamide resistance in 55.6% of pyrazinamide-resistant isolates, and 55% of kanamycin-resistant strains had mutations in the *eis* promoter. We detected no mutations in *alr* and *ddl* genes among cycloserine-resistant isolates, nor in *thyA* gene among paraaminosalicylic acid–resistant isolates.

**Table:** Mutations associated with drug resistance detected in *Mycobacterium tuberculosis* genomes

Tuberculosis drug	Gene variant	No. strains with confirmed drug resistance	Drug-resistant strains, %
Isoniazid	*katG* S315T	52	100
	*fabG-inhA*-15	1	
	*katG* S315T	2	
	*fabG-inhA*-15	2	
Rifampin	*rpoB* S450L	40	100
	*rpoB* D435V	2	
	*rpoB* H445D	1	
	*rpoB* H445R	1	
	*rpoB* D574E	1	
Streptomycin	*rpsL* K43R	33	96.4
	*rpsL* K88R	5	
	*gid* G48G	1	
	*gid* G34G	1	
	*rrs*516	12	
Ofloxacin	*gyrA* D94N	1	81.8
	*gyrA* D94Y	1	
	*gyrA* D94G	5	
	*gyrA* D94A	2	
Ethambutol	*embA*-16	4	72.7
	*embA*-8	1	
	*embB* M3061	2	
	*embB* S3471	1	
	*embB* N399T	1	
	*embB* G406D	2	
	*embB* G406A	2	
	*embB* A453A	1	
	*embB* Q497R	2	
Pyrazinamide	*rpsA* D123A	1	55.6
	*rpsA* A412V	1	
	*pncA* L159R	1	
	*pncA* C138R	1	
	*pncA* T135P	1	
	*pncA* V130E	1	
	*pncA* Q122Stop	1	
	*pncA* Y103Stop	2	
	*pncA* G97S	1	
Kanamycin	*eis*-37	4	55.0
	*eis*-14	3	
	*eis*-10	4	

Our analysis for small insertions and deletions detected 15 and 9, respectively, among the Beijing group (Technical Appendix Table 2). A deletion in *kdpD* and an insertion in *Rv1258c* were previously described (*12**,**13*). The other 22 mutations are novel: 18 were specific to the Beijing group; 2 to the modern Beijing group; 1 to the ancient Beijing group; and 3 to the B0/W148 group. We identified most mutations in genes encoding membrane-associated proteins, although several mutations were in regulatory genes, genes involved in cell metabolism, probable transposase genes, and genes with unknown function.

One insertion and 2 deletions were significantly associated with B0/W148 genetic group in *kdpD, mmr* and Rv1995 (p = 2.5 × 10^−17^) genes. Merker et al. (*12*), who proposed a pathogenic influence for B0/W148 strains, described a frameshift deletion in *kdpD* among Beijing B0/W148 strains. Deletion in the *kdpD* gene can lead to the formation of nonfunctional proteins KdpD and KdpE. Parish et al. showed that *M. tuberculosis* lacking KdpD and KdpE function express increased virulence in a mouse model of infection (*14*), which supports that the *kdpD* deletion detected in our study may influence Beijing B0/W148 strain’s rapid expansion and virulence. A mutation in the promoter region of the *mmr* gene (*Rv3065*), encoding multidrug-transport integral membrane protein, might contribute to drug resistance in Beijing B0/W148 strains. Sriraman et al. recently showed that *mmr* is upregulated in rifampin-resistant and MDR TB strains, even in the presence of target gene mutations (*15*). Insertion in *Rv1258c* is common to all Beijing strains except ancient TB0010. In their study, Villellas et al. found the cytosine nucleotide insertion between positions 580 and 581 in the *Rv1258c* gene in all Beijing isolates among streptomycin-resistant *M. tuberculosis* strains (*13*).

In conclusion, we examined the phylogenetic and drug-resistance properties of *M. tuberculosis* isolates collected from 71 TBS patients in 32 locales across Russia. Our analyses confirmed the phylogenetic separation of pathogenic *M. tuberculosis* strains and support the prevalence of Beijing strains showing high levels of multidrug resistance among TBS isolates. Further, we found known SNP variants that had high concordance with suggested drug resistance. Finally, novel insertions/deletions were apparent, which we suggest are candidates for conferring drug resistance pending independent replication studies. Our analysis of WGS data identified known and novel genetic determinants that could or do influence bacterial virulence, pathogenicity, and drug resistance.

Technical Appendix *Mycobacterium tuberculosis* isolate data; insertions and deletions associated with *M. tuberculosis* genetic clades.
