# Supplementary material for: Whole-Genome Analysis of Mycobacterium tuberculosis from Patients with Tuberculous Spondylitis, Russia
Source: Emerg Infect Dis. 2018 Mar;24(3):579–83. doi: 10.3201/eid2403.170151 (PMC5823328; doi:10.3201/eid2403.170151)
Supplement: Technical Appendix — Mycobacterium tuberculosis isolate data; insertions and deletions associated with M. tuberculosis genetic clades. [file 17-0151-Techapp-s1.pdf]

Article DOI: https://doi.org/10.3201/eid2403.170151

Genome-wide Analysis of *Mycobacterium tuberculosis* from Patients with Tuberculous Spondylitis, Russia

Technical Appendix

Technical Appendix Table 1. *Mycobacterium tuberculosis* isolate data

Strain No.	Major lineage	Genotype/ PhyTB Barcode	SpoTyping SIT	Verified SIT (if available)	Geographic region	HIV status	Year of isolate extraction	SM	INH	RIF	ETH	EMB	KM	OFL	PAS	CS	CM	AM	PZA
TB0002	4	Ural/4.2.1	777	ND	St. Petersburg	–	2011	S	S	S	S	R	S	S	S	S	S	S	S
TB0004	2	Beijing (B0/W148)/ 2.2.1	1	1	St. Petersburg	+	2011	S	S	S	S	S	S	S	S	S	S	S	S
TB0005	4	LAM/4.3.3	ND	ND	Leningrad Region	+	2011	S	S	S	S	S	S	S	S	S	S	S	S
TB0006	2	Beijing/2.2.1	1	1	Chelyabinsk Region	–	2011	R	R	R	R	R	S	S	S	S	S	S	R
TB0008	4	Ural/4.2.1	262	262	Kalmykia	–	2009	R	R	R	R	R	S	S	S	S	S	S	R
TB0009	2	Beijing/2.2.1	1	1	Kaliningrad Region	+	2011	R	R	R	R	S	S	R	S	S	R	R	R
TB0010	2	Beijing/2.2.1	269	269	Buryatia	–	2010	R	R	R	S	S	ND	S	ND	ND	S	S	R
TB0011	2	Beijing (B0/W148)/ 2.2.1	1	1	Volgograd Region	–	2010	R	R	R	R	R	R	S	S	S	R	R	R
TB0012	2	Beijing/2.2.1	1	1	Arkhangelsk Region	–	2010	R	R	R	R	S	R	S	S	S	S	S	R
TB0025	2	Beijing (B0/W148)/ 2.2.1	1	1	Leningrad Region	+	2011	R	R	R	R	R	R	S	S	S	R	R	S
TB0027	2	Beijing/2.2.1	1	ND	Novgorod Region	+	2010	S	S	S	S	S	S	S	S	S	S	S	S
TB0029	4	T/4.8	53	53	Ulyanovsk Region	+	2011	S	S	S	S	S	S	S	ND	ND	R	S	S
TB0032	2	Beijing/2.2.1	1	ND	Zabaykalsky Krai	+	2012	R	R	R	S	S	S	S	ND	ND	S	S	R

Strain No.	Major lineage	Genotype/ PhyTB Barcode	SpoTyping SIT	Verified SIT (if available)	Geographic region	HIV status	Year of isolate extraction	SM	INH	RIF	ETH	EMB	KM	OFL	PAS	CS	CM	AM	PZA
TB0033	4	Ural/4.2.1	1050	1050	Kalmykia	–	2011	S	S	S	S	S	S	S	S	S	S	ND	ND
TB0034	2	Beijing (B0/W148)/ 2.2.1	1	1	St. Petersburg	–	2011	R	R	R	R	R	R	S	R	S	R	R	R
TB0035	2	Beijing (B0/W148)/ 2.2.1	1	1	Leningrad Region	+	2010	R	R	R	R	S	S	S	S	S	S	S	R
TB0036	4	Ural/4.2.1	1134	ND	Leningrad Region	–	2011	R	R	R	S	S	S	S	R	S	S	S	ND
TB0037	2	Beijing (B0/W148)/ 2.2.1	1	1	Kabardino-Balkaria	–	2011	R	R	R	R	R	R	R	S	S	S	S	R
TB0038	2	Beijing/2.2.1	1	1	Amur Region	–	2010	R	R	S	R	S	R	S	S	S	R	R	ND
TB0039	2	Beijing/2.2.1	1	1	Kaluga	–	2009	R	R	S	R	R	R	S	S	S	ND	ND	S
TB0040	2	Beijing/2.2.1	1	ND	Samara	–	2012	R	R	R	R	S	R	S	S	S	S	R	R
TB0041	4	LAM/4.3.3	42	42	Leningrad Region	–	2008	R	R	S	S	R	S	S	R	S	S	S	S
TB0042	4	T/4.8	53	53	Vladimir Oblast	–	2012	R	R	R	S	R	S	S	S	S	R	S	R
TB0043	4	T/ 4.1.2.1	37	ND	St. Petersburg	–	2008	S	S	S	S	S	S	S	ND	ND	ND	ND	ND
TB0044	2	Beijing (B0/W148)/ 2.2.1	1	1	North Osetia	–	2011	R	R	R	S	S	R	S	R	S	S	S	R
TB0045	2	Beijing/2.2.1	1	1	Leningrad Region	–	2008	R	R	R	R	S	R	S	S	S	S	ND	S
TB0046	2	Beijing/2.2.1	1	1	St. Petersburg	–	2009	S	S	S	S	S	S	S	ND	ND	ND	ND	ND
TB0047	4	T/ 4.1.2.1	334	ND	Udmurtya	–	2008	S	S	S	S	S	S	S	S	S	ND	ND	ND
TB0048	2	Beijing/2.2.1	1	ND	St. Petersburg	–	2007	R	R	S	R	S	S	S	S	S	ND	ND	ND
TB0049	2	Beijing (B0/W148)/ 2.2.1	1	ND	St. Petersburg	–	2007	R	R	R	S	R	S	S	S	S	ND	ND	ND
TB0050	2	Beijing (B0/W148)/ 2.2.1	1	ND	Samara	–	2007	R	R	R	S	S	R	S	S	S	ND	ND	ND
TB0051	2	Beijing/2.2.1	1	ND	Kostroma	–	2009	R	R	S	S	S	S	S	S	S	S	ND	ND
TB0052	2	Beijing/2.2.1	1	ND	Leningrad Region	+	2009	R	R	R	R	S	R	S	S	S	S	S	blank
TB0053	2	Beijing/2.2.1	1	ND	St. Petersburg	–	2008	S	S	S	S	S	S	S	ND	ND	ND	ND	ND
TB0054	2	Beijing (B0/W148)/ 2.2.1	1	ND	Khabarovsk	–	2007	R	R	R	S	R	S	S	R	S	ND	ND	ND
TB0055	4	T/ 4.1.2.1	53	ND	Pskov	–	2010	R	R	R	S	R	S	S	S	S	ND	ND	ND

Strain No.	Major lineage	Genotype/ PhyTB Barcode	SpoTyping SIT	Verified SIT (if available)	Geographic region	HIV status	Year of isolate extraction	SM	INH	RIF	ETH	EMB	KM	OFL	PAS	CS	CM	AM	PZA
TB0057	2	Beijing/2.2.1	1	ND	Samara	–	2013	R	R	R	S	R	S	R	S	S	S	S	R
TB0058	2	Beijing/2.2.1	1	ND	Moscow Region	–	2013	R	R	R	S	S	S	R	S	S	S	S	ND
TB0059	2	Beijing/2.2.1	1	ND	Kaliningrad Region	–	2013	R	R	R	R	R	S	S	S	S	S	S	S
TB0061	2	Beijing/2.2.1	1	ND	Chelyabinsk Region	–	2013	R	R	R	S	S	S	S	S	S	S	S	ND
TB0062	2	Beijing/2.2.1	1	ND	Chelyabinsk Region	–	2013	R	R	R	S	S	S	S	S	S	S	S	ND
TB0063	2	Beijing (B0/W148)/ 2.2.1	1	ND	North Osetia	–	2013	R	R	S	R	S	S	S	S	S	S	S	ND
TB0064	2	Beijing/2.2.1	1	ND	Vologda Region	–	2013	S	R	S	S	S	S	S	S	S	R	R	S
TB0065	2	Beijing/2.2.1	1	ND	Leningrad Region	–	2013	R	R	R	S	S	R	S	S	S	S	S	S
TB0066	2	Beijing/2.2.1	1	ND	St. Petersburg	–	2013	R	R	R	R	R	S	S	S	S	S	S	S
TB0067	2	Beijing (B0/W148)/ 2.2.1	1	ND	Leningrad Region	+	2013	R	R	R	S	S	R	S	S	S	S	S	S
TB0068	2	Beijing (B0/W148)/ 2.2.1	1	ND	Altaysky Krai	–	2013	R	R	R	R	S	R	S	S	S	R	R	ND
TB0072	2	Beijing/2.2.1	1	ND	Samara	–	2014	R	R	R	S	S	S	S	R	S	S	S	ND
TB0073	2	Beijing (B0/W148)/ 2.2.1	1	ND	Tatarstan	+	2014	R	R	R	S	S	S	S	S	S	S	S	ND
TB0074	2	Beijing/2.2.1	1	ND	St. Petersburg.	+	2014	R	R	R	R	S	R	S	S	S	R	R	R
TB0075	2	Beijing/2.2.1	1	ND	St. Petersburg.	+	2014	S	S	S	S	S	S	S	S	S	S	S	ND
TB0076	4	T /4.8	52	ND	Perm Krai	–	2014	S	S	S	S	S	S	S	S	S	S	S	ND
TB0077	2	Beijing (B0/W148)/ 2.2.1	1	ND	Leningrad Region	+	2014	R	R	R	S	R	R	R	R	S	R	R	ND
TB0078	2	Beijing (B0/W148)/ 2.2.1	1	ND	Tver Oblast	+	2014	R	R	R	R	S	S	R	R	S	R	S	ND
TB0079	2	Beijing (B0/W148)/ 2.2.1	1	ND	Kursk Region	–	2014	R	R	S	R	S	S	S	R	S	S	S	ND

Strain No.	Major lineage	Genotype/ PhyTB Barcode	SpoTyping SIT	Verified SIT (if available)	Geographic region	HIV status	Year of isolate extraction	SM	INH	RIF	ETH	EMB	KM	OFL	PAS	CS	CM	AM	PZA
TB0080	2	Beijing (B0/W148)/ 2.2.1	1	ND	Bryansk Region	–	2014	R	R	R	R	R	S	S	S	S	S	S	R
TB0081	4	T/4.8	131	ND	Leningrad Region	–	2014	S	S	S	S	S	S	S	S	S	S	S	ND
TB0083	2	Beijing/2.2.1	1	ND	Altaysky Krai	–	2014	R	R	R	R	R	S	S	S	S	S	S	ND
TB0084	2	Beijing (B0/W148)/ 2.2.1	1	ND	Altaysky Krai	+	2014	R	R	R	S	S	R	S	R	S	S	R	ND
TB0085	2	Beijing/2.2.1	1	ND	Leningrad Region	–	2014	R	R	R	S	S	S	S	S	S	S	S	ND
TB0086	2	Beijing/2.2.1	1	ND	Buryatia	–	2014	S	S	S	S	S	S	S	S	S	S	S	ND
TB0087	2	Beijing (B0/W148)/ 2.2.1	1	ND	Irkutsk Region	+	2014	R	R	R	S	S	S	S	R	S	S	S	ND
TB0088	2	Beijing/2.2.1	1	ND	Samara	–	2014	R	R	R	S	S	S	R	S	S	S	S	ND
TB0089	2	Beijing/2.2.1	1	ND	Leningrad Region	+	2014	R	R	R	S	R	R	S	R	S	R	S	ND
TB0092	2	Beijing (B0/W148)/ 2.2.1	1	ND	Chelyabinsk Region	–	2014	R	R	R	R	S	R	R	S	S	S	S	ND
TB0093	2	Beijing/2.2.1	1	ND	Arkhangelsk Region	+	2014	R	S	S	S	S	S	S	S	S	S	S	ND
TB0094	2	Beijing/2.2.1	1	ND	Moscow Region	+	2014	R	R	R	R	R	R	R	S	S	S	S	ND
TB0095	2	Beijing/2.2.1	1	ND	Buryatia	+	2014	R	R	R	S	S	S	S	S	S	S	S	ND
TB0098	2	Beijing/2.2.1	1	ND	Zabaykalsky Krai	–	2014	S	S	S	S	S	S	S	S	S	S	S	S
TB0113	2	Beijing/2.2.1	1	ND	Orenburg Region	–	2014	R	R	R	R	R	S	R	S	S	S	S	R
TB0157	2	Beijing/2.2.1	1	ND	Dagestan	–	2014	R	R	R	S	S	R	S	S	S	R	R	R

*Phy TB, phylogenic analysis of Mycobacterium tuberculosis; spo, spondylitis; SIT, strains isolated in patients; HIV, human immunodeficiency virus; SM, streptomycin; INH, isoniazid; RIF, rifampicin; ETH, ethionamide; EMB, ethambutol; KM, kanamycin; OFL, ofloxacin; PAS, para-aminosalicylic acid; CS, cycloserine; CM, capreomycin; AM, amikacin; PZA, pyrazinamide; + positive; – negative; S ,drug susceptible; R, drug resistant; ND, no data.

Technical Appendix Table 2. Insertions and deletions associated with *M. tuberculosis* genetic clades

Isolate no.	Genetic clade	Gene position	Genome coordinates	Reference sequence	Alternative sequence	Effect	Genome region	Gene name
1	Beijing	682	99162	TC	TCGGTGTGCGC	Deletion	CDS	Rv0090
2	Beijing	511	194305	CGGC	CC	Deletion	CDS	mce1R
3	Beijing	-56	507028	GCCCCCG	GCCCCCCG	Insertion	Intergenic	Rv0420c
4	Beijing	-12	1090188	AGGG	AGG	Deletion	Intergenic	Rv0976c
5	Beijing	336	1168009	GCCCCG	GCCCCCG	Insertion	CDS	Rv1045
6	Beijing	581	1406760	TGC	TC	Deletion	CDS	Rv1258c
7	Beijing	96	1413129	CGGGGGC	CGGGGGGC	Insertion	Non-coding RNA	mcr11
8	Beijing	118	2009290	GCCCCA	GCCCCCA	Insertion	CDS	Rv1775
9	Beijing	872	2241031	AG	AGG	Insertion	CDS	ctpF
10	Beijing	841	2342649	AGGCGTACACACG	AG	Deletion	CDS	Rv2084
11	Beijing	105	2734481	TC	TAC	Insertion	CDS	Rv2437
12	Beijing	53	3021892	GTTCGACGACT	GTTCGACGACTTCGACGACT	Insertion	CDS	Rv2709
13	Beijing	1,308	3194241	CGGC	CGC	Deletion	CDS	Rv2885c
14	Beijing	381	3238119	CGTGC	CGTGGTGC	Insertion	CDS	Rv2923c
15	Beijing	1	3794867	CCAC	CC	Deletion	CDS	dxs2
16	Beijing	-19	4221070	AC	ACC	Insertion	Intergenic	Rv3776
17	Beijing	623	4305063	GAAA	GAA	Deletion	CDS	Rv3830c
18	Beijing	501	4322039	AC	ACC	Insertion	CDS	Rv3847
19	Beijing B0/W148	2,545	1149142	CTGT	CT	Insertion	CDS	kdpD
20	Beijing B0/W148	720	2238861	GAAAAAAG	GAAAAAG	Insertion	CDS	Rv1995
21	Beijing B0/W148	-29	3430358	CATGTACAAA	CATGTACAAATGTACAAA	Deletion	Intergenic	mmr
22	Modern Beijing	91	1418863	CGGGAGCCAG	CGGGAGCCAGGGAGCCAG	Deletion	CDS	Rv1269c
23	Modern Beijing	633	2703902	CGGC	CGC	Deletion	Intergenic	Rv2405
24	Ancient Beijing	-106	809840	GCCCCCCA	GCCCCCCCA	Deletion	Intergenic	Rv0713
